# Multi-scale multi-physics model of brain interstitial water flux by transcranial Direct Current Stimulation

**DOI:** 10.1088/1741-2552/ace4f4

**Published:** 2023-07-24

**Authors:** Niranjan Khadka, Cynthia Poon, Limary M Cancel, John M Tarbell, Marom Bikson

**Affiliations:** 1 Synchron Inc, New York, United States of America; 2 Department of Biomedical Engineering, The City College of New York, CUNY, New York, United States of America

**Keywords:** blood brain barrier (BBB), neurovascular-modulation, neurovascular coupling, electroosmosis, interstitial water flux, transcranial direct current stimulation (tDCS)

## Abstract

**Objective.:**

Transcranial direct current stimulation (tDCS) generates sustained electric fields in the brain, that may be amplified when crossing capillary walls (across blood-brain barrier, BBB). Electric fields across the BBB may generate fluid flow by electroosmosis. We consider that tDCS may thus enhance interstitial fluid flow.

**Approach.:**

We developed a modeling pipeline novel in both (1) spanning the mm (head), μm (capillary network), and then nm (down to BBB tight junction (TJ)) scales; and (2) coupling electric current flow to fluid current flow across these scales. Electroosmotic coupling was parametrized based on prior measures of fluid flow across isolated BBB layers. Electric field amplification across the BBB in a realistic capillary network was converted to volumetric fluid exchange.

**Main results.:**

The ultrastructure of the BBB results in peak electric fields (per mA of applied current) of $32–63\;V{\;m}^{-1}$ across capillary wall and $>1150\;V\;m^{-1}$ in TJs (contrasted with $0.3\;V\;m^{-1}$ in parenchyma). Based on an electroosmotic coupling of $1.0\times 10^{-9}-5.6\times 10^{-10}\frac{m^{3}\;s^{-1}}{\;m^{2}}$ per $Vm^{-1}$, peak water fluxes across the BBB are $2.44\times 10^{-10}-6.94\times 10^{-10}\frac{m^{3}\;s^{-1}}{\;m^{2}}$, with a peak $1.5\times 10^{-4}-5.6\times 10^{-4}\frac{m^{3}\;{min}^{-1}}{\;m^{3}}$ interstitial water exchange (per mA).

**Significance.:**

Using this pipeline, the fluid exchange rate per each brain voxel can be predicted for any tDCS dose (electrode montage, current) or anatomy. Under experimentally constrained tissue properties, we predicted tDCS produces a fluid exchange rate comparable to endogenous flow, so doubling fluid exchange with further local flow rate hot spots (‘jets’). The validation and implication of such tDCS brain ‘flushing’ is important to establish.

## 1. Introduction

Transcranial direct current stimulation (tDCS) is brain stimulation from passing low-intensity (few mA) current between electrodes on the scalp (Woods et al 2016). tDCS generates brain electric fields of $∼0.5\;V\;m^{-1}$ per mA of applied current (Datta et al 2009, Huang et al 2017), which polarize neuronal compartments (Rahman et al 2013), associated with changes in function (Stagg et al 2018). Diverse applications of tDCS are based on modulation of neuronal excitability including clinical trials in Alzheimer’s disease (AD) and related dementias (Narita and Yokoi 2017, Tsapkini et al 2018, da Silva et al 2022, Pilloni et al 2022, Martorella *et al* 2023a), as well as remediation of cognitive decline in healthy older adults (Arciniega et al 2018, Cruz Gonzalez et al 2018, Ljubisavljevic et al 2019, Indahlastari et al 2021, Iordan et al 2022).

Neurovascular-modulation is a mechanism of action that considers direct vascular and blood brain barrier (BBB) stimulation, leading to secondary changes in neuronal function (Khadka and Bikson 2020, Bahr-Hosseini and Bikson 2021). Hemodynamic based imaging (e.g., fMRI, fNIRS) of tDCS (Zheng et al 2011, Jamil et al 2020, Lu et al 2020, McKendrick et al 2020, Nakashima et al 2021) may thus reflect direct vascular stimulation. Studies in animal models provide an explanation for tDCS neurovascular-modulation whereby (1) electric fields are amplified (∼400×) across the BBB to $200\;V\;m^{-1}$; (2) direct electric fields across the BBB, and specifically tight junctions (TJs), generate water flux by electroosmosis (Cancel et al 2018) which; (3) in turn modulate solute transport (Cancel et al 2018, Shin et al 2020), blood flow (Wachter et al 2011, Mielke et al 2013, Gellner et al 2023), and changes in BBB ultrastructure function (Xia et al 2021); as well as (4) solute diffusivity across brain tissue (Xia et al 2020).

Electroosmosis thus serves as the biophysical coupling between electrical stimulation and enhanced brain transport. Electroosmotic flow arises from the movement of charged ions through channels that have fixed charge on their surfaces (Grodzinsky 2011). Cell membranes present a fixed negative surface charge, including in the TJs formed between endothelial cells of the BBB (Li and Fu 2011). In response to an applied electric field, a layer of net positive mobile charge near the fixed charge can move, dragging with it the surrounding fluid. Thus, electric fields generate fluid transport through the channel. The relevance of electroosmosis to overall transport is magnified in very small channels where pressure-driven convection is limited by hydrodynamic resistance, as the case for the BBB (Cancel et al 2018). The amount of fluid flow will depend on the dose of electrical stimulation, how brain anatomy and vasculature structure then govern the electric fields produced across the BBB, and the coupling of electric field with fluid flow by electroosmosis.

The therapeutic implications of tDCS neurovascular-modulation (Bikson 2023) can include: (1) changes in brain vascular function that can broadly modulate neuronal function (Daffertshofer and Hennerici 1995, Moore and Cao 2008); (2) Restoration of cerebral blood flow which is of broad interest in brain disorders (Matsuda 2001); and (3) Enhancement of water flux and solute transport that may boost brain clearance, that may be impaired in AD and other brain disorders (Tarasoff-Conway et al 2015).

The purpose of this study is to enhance computational models of BBB (capillary wall) polarization by tDCS, couple this polarization by electroosmosis to fluid flow model across the BBB, and then predict interstitial water exchange. To this end, we developed a modeling pipeline simulating current flow down through three-scales (from anatomy (millimeter) to vessels (micrometer) to TJ (nanometer)) and then simulated fluid flow up these scales. Subject to experimentally constrained parameters, we predicted tDCS can double the rate of fluid exchange in the brain, and moreover produce local ‘jets’ of flow. This prediction supports and provides a quantitative basis for approaches to treat brain disease by tDCS neurovascular-modulation.

## 2. Methods

### 2.1. Overview of multi-scale multi-physics tDCS modeling pipeline

**Figure 1.**
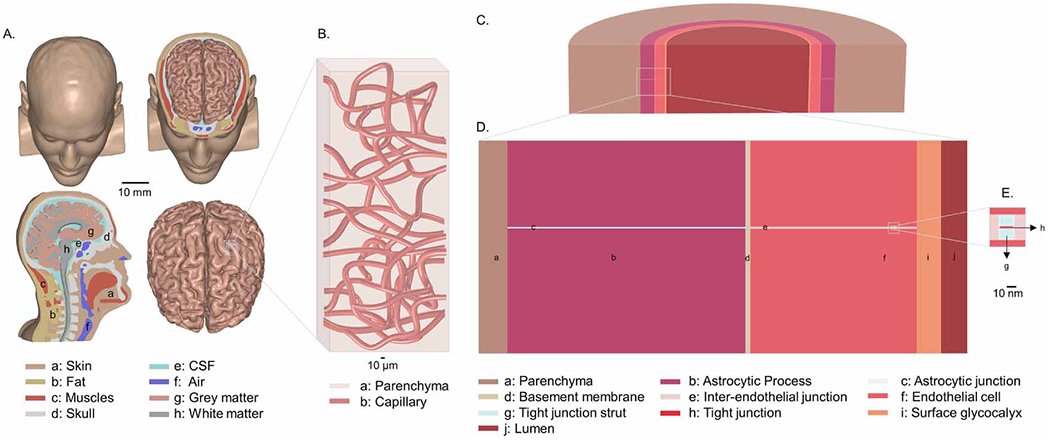
Multi-scale anatomical representation of brain, vasculature, and blood brain barrier model. (A) represents an anatomically realistic template human head model showing segmented brain and other tissue-compartments in millimeter scale (macroscopic level). (B) shows micrometer scale (capillary level) representation of realistic CAD-derived illustration of capillary network including capillary wall (1μm thick) and lumen, derived from imaging study (Secomb et al 2004, Celaya-Alcala et al 2021) into a brain region of interest (ROI). (C) shows a 3D sectional view of a nanometer scale (tight junction level: 2 nm thick) model of BBB ultrastructure. (D) and (E) illustrate cross sectional view and inset of the BBB ultrastructure showing brain, astrocytic process, astrocytic junction, basement membrane, endothelial cell, tight junction channel, tight junction cleft, tight junction, surface glycocalyx, and lumen. The dimensions of the BBB ultrastructures were based on prior studies (Adamson et al 2004, Shin et al 2020).

**Figure 2.**
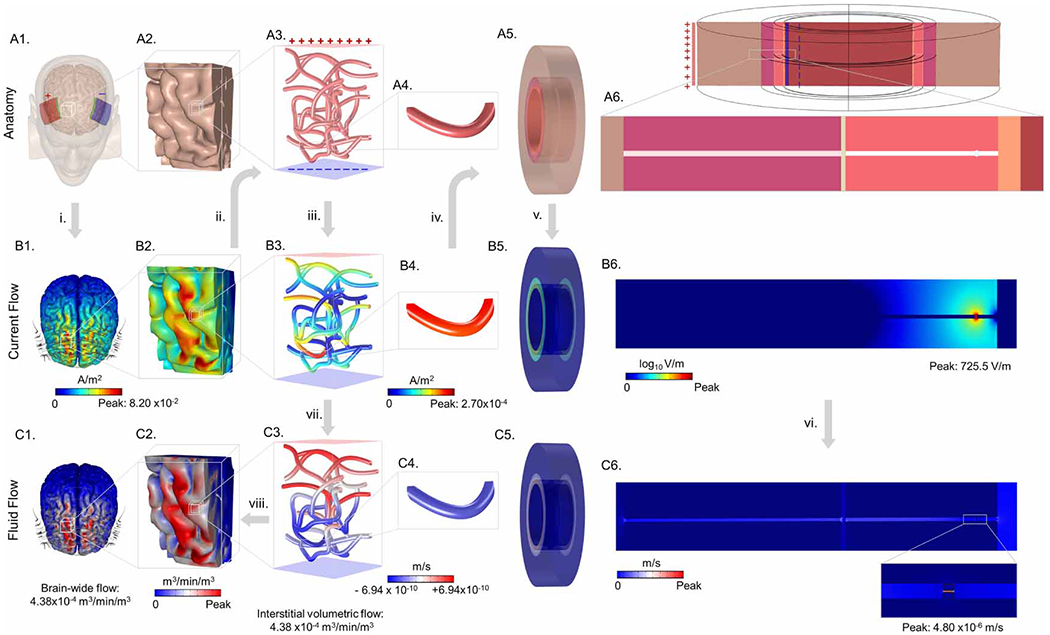
Multi-scale multi-physics modeling pipeline of tDCS-coupled water flux using an exemplary vasculature network. The top rows show anatomical representation of millimeter (brain), micrometer (capillary network), and nanometer (tight junction) scale of model. The middle row represents predicted current density and electric field profile in all scales of model. The third row shows fluid flow rate across tight junction, BBB, and brain. Specifically, (A1) represents a bifrontal montage of tDCS, (A2) a region of interest (ROI) of brain where vasculature network was derived (A3), (A4) zoomed in cutout of a capillary network loop of A3, (A5) 3D BBB ultrastructure model derived from blood vessel cross-section with circumferential symmetry, (A6) cross-sectional view of A5 including tight junction. (B1) and (B2) show predicted current flow pattern in the brain, (B3) and (B4) across the vasculature, and (B5) & (B6) shows current density and electric field at the BBB ultrastructure. (C1) and (C2) represent brain-wide fluid exchange, (C3) and (C4) across BBB, and (C5) and (C6) across BBB ultrastructure, including tight junction. The modeling pipeline (shown by arrows with roman letters) first predicted peak current density at the millimeter scale (brain), micrometer scale (capillary), nanometer scale (tight junction), and applied peak current density at each level of model as boundary condition for the following model scale. Across model scales, there was an amplification of current density. Next, the pipeline then calculated tDCS-induced fluid flow starting at the nanoscale, through the capillary scale, and up to brain-wide fluid exchange. Generally, step (i) shows tDCS included brain current flow. Step (ii) shows peak brain ROI-specific current density applied as a boundary condition for the capillary network. Step (iii) shows predicted current density across BBB macroscopic structure. In step (iv), peak current density across the BBB was applied as an input for the BBB ultrastructure model. Step (v) shows predicted electric field at the BBB ultrastructure. Step (vi) is an illustration of tight junction fluid flow by the electric field called electroosmotic flow. In step (vii), the volumetric fluxes per nodes (inward and outward) across the vascular network was first multiplied by the vessel area per nodes, and then divided by the volume of the voxel containing the capillary network to get the net interstitial volumetric fluid exchange. Finally, in step (viii), the capillary level volumetric interstitial flow was translated to brain-wide fluid exchange by tDCS.

We developed a three-scales finite element method model starting with: (1) a realistic MRI-derived brain anatomy (millimeter scale) (figures 1(A) and 2(A1), (A2)), then (2) adapted a scanning electron microscopy (SEM) realistic microvasculature network into a brain region of interest (ROI) (micrometer scale) (figures 1B and 2(A3), (A4)), and (3) finally developed a BBB ultrastructure model (nanometer scale) (figures 1(C)–(E) and 2(A5), (A6)) including a TJ from an exemplary vasculature cross-section. Across scales, a ROI was defined based on peak predicted current density. The modeling pipeline first calculated current flow patterns at the millimeter (head) scale (figures 2(B1) and B2), micrometer (capillary) scale (figures 2(B3) and (B4)), and nanometer (BBB ultrastructure) scale (figures 2(B5) and (B6)), where the result from each stage (peak current density) was used as an input (boundary current density) to the next stage. Thus, current density amplification down each scale was simulated. Current flow was then coupled to fluid flow by electroosmosis at TJs. Fluid flow was then simulated up each scale to predict interstitial volumetric flow per brain volume. The modeling pipeline thus calculated fluid flow starting at the nanoscale (figures 2(C5) and (C6)), through the capillary scale (figures 2(C3) and (C4)), and back up to brain wide fluid exchange (figures 2(C1) and (C2)).

### 2.2. Millimeter scale brain model architecture and parameters

An anatomical model of a human head (figure 1(A)) based on an optimized high-resolution $\left(1\;{mm}^{3}\right)$ MRI scans was developed and segmented into homogeneous tissue compartments representing scalp/skin, fat, skull, cerebrospinal fluid (CSF)/meninges, gray matter, and white matter using a series of automatic and manual segmentation algorithms of Simpleware (Synopsys, CA, USA). A computer aided design (CAD) model of $5\;\times \;7\;{cm}^{2}$ rectangular sponge-electrodes (1mm thickness) was modeled in Solidworks (Dassault Systemes Corp., MA, USA) and later imported and positioned over the cortex in a bilateral or M1-SO configuration using Simpleware. The volumetric mesh generated from a voxel based tetrahedral adaptive meshing algorithm was exported in COMSOL Multiphysics 5.5 (COMSOL Inc., MA, USA) to computationally solve the current density profile (Seibt et al 2019).

The resulting mesh across for the simulated montage comprised >12M tetrahedral elements. Electrical conductivity of the brain tissues and electrodes were assigned as: scalp/skin: $0.465\;S\;m^{-1}$, fat: $0.025\;S\;m^{-1}$, skull: $0.01\;S\;m^{-1}$, CSF/meninges: $0.8\;S\;m^{-1}$, gray matter: $0.276\;S\;m^{-1}$, white matter: $0.126\;S\;m^{-1}$, and anode/cathode electrodes: $5.9\times 10^{7}\;S\;m^{-1}$ (Jiang et al 2020).

### 2.3. Micrometer scale realistic microvasculature network architecture and parameters

A realistic 3D microvascular network model (figures 1(B) and 4, 5) was developed adapting node-based geometric data reconstructed from SEM of corrosion casts (Secomb et al 2004, Celaya-Alcala et al 2021). The final capillary CAD network was constructed in SolidWorks using a built-in spline function. Capillaries with a wall thickness of 1μm and an outer diameter ranging from 8 to 10μm were constructed within a brain voxel of $0.15\;\times \;0.16\;\times \;0.43\;{mm}^{3}$. The length, surface area, and volumetric densities for the capillary network, approximating *in vivo* data (Schlageter et al 1999, Secomb et al 2004, Kreczmanski et al 2009, Ashraf et al 2020, Khadka and Bikson 2020, Celaya-Alcala et al 2021) were $557\;mm\;{mm}^{-3},22.5\;{mm}^{2}\;{mm}^{-3}$, and 0.021, respectively.

The final realistic microvascular network model was imported into COMSOL Multiphysics 5.5 and assigned standard isotropic electrical conductivities to the tissue compartments as: brain parenchyma: $0.276\;S\;m^{-1}$, capillary wall: $1\;\times \;10^{-5}\;S\;m^{-1}$ (1000 transendothelial electrical resistance (TEER)) and $2\;\times \;10^{-6}\;S\;m^{-1}$ (5000 TEER), and capillary lumen: $0.7\;S\;m^{-1}$. The TEER is defined in detail below. The final volumetric mesh generated using a built-in adaptive meshing algorithm comprised >10M tetrahedral elements.

The conductivity of the capillary wall was estimated from the vessel TEER. The conductivity of the vessel wall was assumed to be uniform for the micrometer scale realistic network model. In this case, the resistivity, $\rho \left(\Omega^{*}\;m\right)$, of the wall was estimated from the experimental vessel TEER $\left(\Omega *\;m^{2}\right)$ by:

(1) $$\text{TEER}=R_{\text{wall}}*A_{\text{vessel}}=\rho_{\text{wall}}*\ell_{\text{wall}}$$

where $R_{\text{wall}}(\Omega )$ is the resistance of the wall, $A_{\text{vessel}}\left(m^{2}\right)$ is the surface area of the vessel, $\ell_{\text{wall}}(m)$ is the thickness of the wall, and $\rho_{\text{wall}}\;(S)$ is the resistivity of the wall. The conductivity was calculated as ${\rho_{\text{wall}}}^{-1}$. This micron scale description did not consider the nanoscale architecture of the interendothelial junction.

### 2.4. Nanoscale BBB ultrastructure architecture and parameters

A nanoscale BBB ultrastructures was modeled (figures 1(C)–(E)) in COMSOL comprising parenchyma, astrocyte process, astrocytic channel, basement membrane, endothelial cell, interendothelial cleft (TJ channel), TJ, TJ strut, surface glycocalyx, and lumen (blood). The model was developed as a blood vessel cross-section with circumferential symmetry (figure 1(C)). This idealization assumes that a single endothelial cell was wrapped around the circumference of the capillary and formed interendothelial clefts with adjacent endothelial cells. The structure in figure 1(D) is rotated in the circumferential direction resulting in a single cleft around the capillary circumference. While this was an approximation of the cleft length, in the final calculations, we used the interendothelial cleft length per unit endothelial surface area based on physiological measurements. The dimensions of the BBB ultrastructures were based on prior studies (Adamson et al 2004, Shin et al 2020). Specifically, we modeled a single 20 nm wide interendothelial junction channel with a 11nm×2nm (length × width) TJ positioned at 105nm (15% of the 700 nm TJ channel length, Adamson et al (2004)) away from the surface of a surface glycocalyx.

Electrical properties of the BBB ultrastructures were assigned as: parenchyma: $0.276\;S\;m^{-1}$ (conductive), astrocyte process, endothelial junction side walls: $1\;\times \;10^{-5}\;S\;m^{-1}$ (insulating), astrocytic channel, endothelial junction channel: $1.5\;S\;m^{-1}$, basement membrane, surface glycocalyx: $0.3\;S\;m^{-1}$, and lumen: $0.7\;S\;m^{-1}$ (Khadka and Bikson 2020). The conductivity of the TJ was estimated from the vessel TEER values as outlined below. An adaptive tetrahedral mesh using a built-in voxel-based meshing algorithm was implemented to generate a refined mesh density until additional model refinement produced less than 1% difference in TJ *E*-field. The resulting model consisted of >100M tetrahedral elements with an average element quality of 0.66.

For the nanoscale BBB ultrastructure model, the conductivity of the TJ was estimated from the vessel TEER by assuming that the TJ accounts for all the vessel conductivity (resistivity) such that:

(2) $$\text{TEER}=R_{\text{TJ}}*A_{\text{vessel}}=\frac{\rho_{\text{TJ*}}\ell_{\text{TJ}}}{2h*L_{\text{TJ}}}$$

where $R_{TJ}\;(\Omega )$ is the resistance of the TJ, $\ell_{TJ}(nm)$ is the length of the $TJ(11\;nm),\;L_{TJ}\left(m*m^{-2}\right)$ is the junction length per unit area of vascular wall $\left(150 000\left(\;m*m^{-2}\right)\right)$, and 2h(nm) is the opening of the TJ (2nm). Unless otherwise stated, simulations were run with wall and TJ conductivities corresponding to 1000 TEER $\left(\Omega^{*}\;{cm}^{2}\right)\left(3.67\;\times \;10^{-4}\;S\;m^{-1}\right)$. In some simulations, TEER value was increased to 5000 $\left(7.34\;\times \;10^{-5}\;S\;m^{-1}\right)$).

### 2.5. Multi-scale model solution methods for electrical current

For the MRI-derived head model (mm scale, figure 1(A)) of current flow, an inward normal current density $\left(A\;m^{-2}\right)$ corresponding to 1 mA bifrontal tDCS (unless otherwise stated) and applied to the exposed surface of the anode sponge-electrode with ground applied to the exposed surface of the cathode electrode. The remaining external boundaries of the head model were electrically insulated (figure 2(A1)). The current density across the brain was simulated (figures 2(A1)–(B1), arrow ‘i’). All subsequent steps and results corresponding to 1 mA tDCS, unless otherwise stated.

For the micrometer scale capillary model of current flow (figure 2(A3)), the peak current density from the head-scale model at the parenchyma (grey matter) was applied as the input boundary condition (inward current density) to the top surface of the brain voxel containing the realistic vasculature network (figures 2(B2) to (A3), arrow ‘ii’). Thus, we simulate a small microvascular network located in a brain voxel that is receiving peak parenchyma current density during tDCS (figures 2(A3), 4(A) and 5(A)).

For the micrometer scale capillary model of current flow (figures 1(B), 4 and 5), two current-return boundary conditions were considered with the realistic micrometer network:

A ‘closed’ vascular network, where locally all current that crosses from the brain into the blood was balanced elsewhere in the vascular network by current exiting from the blood to the brain. In the ‘closed’ network, this was achieved by grounding the bottom surface, with the remaining outer surfaces (the voxel sides) insulated (figure 4(A)). Note that all vessels terminate at these side surfaces, effectively sealing their ends. Thus, current enters from the top surface of the brain voxel, and either travels around the blood vessels, or enters the vessels at one vessel wall surface and then travels through a portion of the lumen of the vascular network and exits at the other vessel wall surface, before all current being finally collected at the bottom surface of the brain voxel. The grounded bottom surface is thus conceptually a current sink to the remainder of the brain and eventually back to the return electrode.

An ‘open’ network assumes some current crossing from brain into local capillaries will be collected through larger vessels to the other brain region. This was achieved in the ‘open’ network by grounding a limited number of the interior surfaces of the vessels, as well as the bottom surface of the brain voxel (figure 5(A)). The remaining external boundaries of the realistic vasculature network model (voxel sides) remained electrically insulated. As a result, current enters from the top surface of the brain voxel, some current enters the vessels at a vessel wall surface, travels through the lumen of the vascular network, until being collected at either: (a) the grounded vessel interiors (representing an unlimited current sink through a larger implicit vascular network) or (b) by exiting the capillary network at another vessel wall at the bottom brain voxel surface.

Note for both ‘closed’ and ‘open’ capillary network, it was possible for some current to cross from the brain into the vessel, exit the vessels at a second location, and then re-enter the vessels at a third location as long as all current ends at the bottom voxel surface (for the closed network) or grounded vessel interiors/bottom brain voxel surface (for the open network). Both the open and closed networks were simulated at 1000 and 5000 TEER, resulting in four total micrometer scale capillary model conditions. In each capillary network model case, the current density produced across all the capillaries walls is predicted (figures 2(A3)–(B3), arrow ‘iii’). Thus, we simulate the peak BBB current density, in the brain voxel receiving peak parenchyma current density, for 1mA frontal tDCS (unless indicated otherwise).

The predicted peak normal current density across the capillary wall (for each capillary network condition) (figure 2(B3)) was used as an input boundary condition for the TJ (nanoscale) current flow computation (figures 2(B4) to (A5), arrow ‘iv’). As the current density was applied to the exterior surface of the blood vessel cross-section (parenchyma outer surface), and the current density was geometry concentrated radially as it arrived at the BBB outer surface, the applied current density was corrected accordingly. Specifically, an adjusted current density (normal capillary wall current density/2.04) according to the area ratio $\left(3.71\;\times \;10^{-10}\;m^{2}/1.57\;\times \;10^{-10}\;m^{2}=2.04\right)$ between the parenchyma and the astro-endothelial outer surfaces was applied to the outer surface of the parenchyma voxel. The outer surface of the lumen (opposite side) was grounded, while the remaining external boundaries of the BBB ultrastructure model was insulated.

To simulate direct current stimulation and predict current density/*E*-field at the brain parenchyma, realistic capillary network, and different BBB ultrastructures, the Laplace equation (Bikson et al 2015) was solved under steady state assumption in all scale models as:

(3) ∇(σ∇V)=0

where V (in V) is an extracellular voltage and σ(S/m) is an electrical conductivity.

### 2.6. Calculation of electroosmotic flow

**Figure 3.**
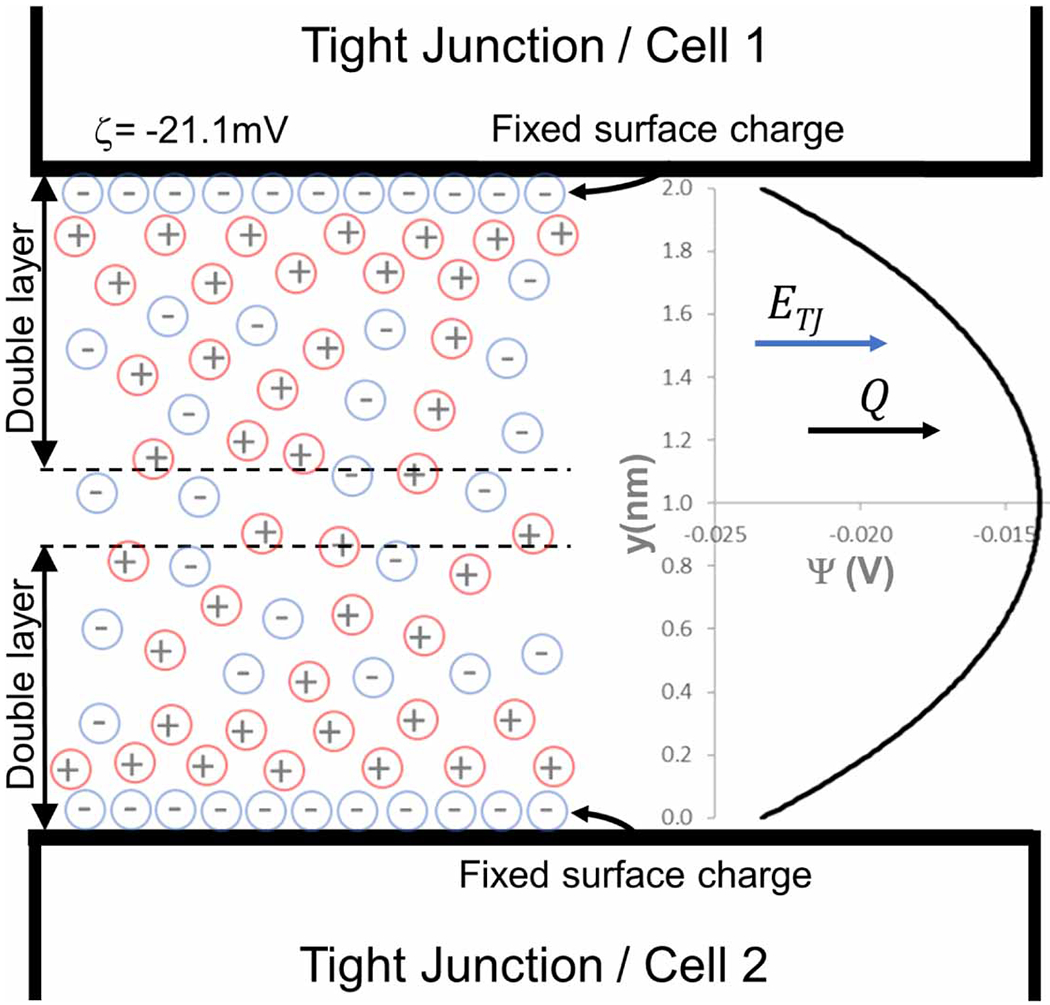
Schematic diagram of the BBB tight junction (TJ) channel with double layers. The negative charges on the endothelial cells surface are fixed, while the surrounding counterions form a diffuse layer (double layer) that can move. The double layer has a net positive charge equal to the net negative charge at the surface. The complete structure is electrically neutral but positive charges are more mobile. Upon application of an external electric field $\left(E_{TJ}\right)$ the excess free positive ions and their bound water move in the direction of the electric field, resulting in a net transport of fluid (Q). The electric potential (ψ; in volts) across a channel of width 2 nm, calculated by equation (4), is shown.

We hypothesized that an electroosmotic flow through the TJ is a source of interstitial flow induced by DCS (Cancel et al 2018); this is central to the coupling between electrical current flow and fluid flow in the new model described here. As in (Cancel et al 2018), we modeled the TJ channel using a simplified structure, proposed by (Fu et al 1994), consisting of two parallel walls forming a narrow slit (figure 3). While this model assumes smooth walls and therefore does not account for the rows of protruding proteins that form the junction channel, our previous study showed excellent agreement between the model and experimental measurements of electroosmotic flow (Cancel et al 2018). In the present study, we have further refined this model by allowing for weak interaction of the double layers, given their proximity in the TJ. Assuming 2D flow in channels with parallel walls and weak double layer overlap, a theoretical approximation of electroosmotic flow was described by Garcia et al (2005) as:

(4) $$\psi (y)=\frac{4kT}{ze}\left[{\text{tanh}}^{-1}\left(\text{tanh}\frac{ze\zeta }{4kT}e^{-\kappa y}\right)\right]\;+\frac{4kT}{ze}\left[{\text{tanh}}^{-1}\left(\text{tanh}\frac{ze\zeta }{4kT}e^{-\kappa (2h-y)}\right)\right]$$

where ψ(y) is an equilibrium potential distribution in the TJ, k is the Boltzmann constant $\left(1.38 064 852\;\times \;10^{-23}\;m^{2}\;kg\;s^{-2}\;K^{-1}\right)$, *T* is the temperature (310K), *z* is the ion valence (1), *e* is the electronic charge $\left(1.60 217 662\;\times \;10^{-19}\;C\right)$, *ζ* is the zeta potential $(-0.0211\;V),\kappa^{-1}$ is the Debye length $\left(9.048 55\;\times \;10^{-10}\;m\right)$, and h is the half-width of the channel $\left(1\;\times \;10^{-9}\;m\right)$. For wide channels the potential is zero everywhere in the channel apart from the narrow double layer near the wall, resulting in a constant electroosmotic velocity. For narrow channels the electrical double layers occupy a great part of the channel width, and the potential is non-zero throughout the channel (figure 3).

The electroosmotic velocity, $u_{eo}(y)$ can then be calculated by Garcia et al (2005) as:

(5) $$u_{\text{eo}}(y)=-\frac{\varepsilon \zeta E}{\mu }\left(1-\frac{\psi (y)}{\zeta }\right)$$

where ε is the permittivity of the medium $\left(7.08\;\times \;10^{-10}\;{F\;m}^{-1}\right)$, μ is the viscosity $\left(7.80\;\times \;10^{-04}\;{Pa}^{*}\;s\right)$, and $E\left(\;V\;m^{-1}\right)$ is the applied E-field intensity.

Equations (4) and (5) were solved numerically to obtain the average velocity in the TJ as a function of the applied E-field, which were related linearly as:

(6) $$v=3.82\;\times \;10^{-9}*E_{\text{TJ}}$$

where $v\left(\;m\;s^{-1}\right)$ is an average fluid velocity and $E_{TJ}\left({V\;m}^{-1}\right)$ is an E-field intensity in the TJ.

Multiplying equation (6) by the width of the TJ and the length of the junction per unit vessel area $\left(L_{TJ}\left(mm^{-2}\right)\right)$ yielded an equation for the volumetric flow rate per unit vessel area $\left(\frac{Q}{A}\right)$, referred to a volumetric flux as:

(7) $$\frac{Q}{A}=1.15\;\times \;10^{-12}*E_{\text{TJ}}$$

where $Q\left(\;m^{3}\;s^{-1}\right)$ is the volumetric flow rate and $A\left(m^{2}\right)$ is the vessel area normal to the flow.

### 2.7. Calculation of volumetric flux across capillary vessels, as driven by electroosmosis, and then net fluid exchange per brain volume

The *E*-field intensity at the TJ can be converted into a volumetric flux using equation (7). To obtain the volumetric flux $\left(\frac{Q}{A}\left(\frac{m^{3}/s}{m^{2}}\right)\right.$ for each location of the capillary wall, the peak current density $\left(J_{v_{i}}\left(\;A\;m^{-2}\right)\right)$ at the capillary wall (figure 2(B3)) was first applied as an input boundary condition for the nanoscale TJ model (arrow ‘iv’), and the E-field intensity at the $TJ\left(E_{TJ}\right.$ was predicted (figures 2(B5) and (B6)). The relationship between $E_{TJ}\left(V\;m^{-1}\right)$ and the $J_{v_{i}}\left(A\;m^{-2}\right)$ at the microcapillary network was then given as:

(8) $$E_{\text{TJ}}=C*J_{v_{i}}$$

where $C\left(\frac{V*\;m}{\;A}\right)$ is a proportionality constant that varies with the resistivity of the TJ (TEER) and the number of TJs. For 1 TJ, the value of C was $2.73\times 10^{6}\frac{V*\;m}{\;A}$ and $3.83\;\times \;10^{6}\frac{V*\;m}{A}$ for 1000 TEER and 5000 TEER, respectively. Note only a single 2 nm thick TJ is modeled here.

Combining equation (7) with equation (8), we obtained a linear relationship (equation (9)) to convert the current density normal to the wall $\left(J_{\text{norm}}\right)$, across each capillary wall segment (figure 2(B3)), into a volumetric flux at that segment (figure 2(C3)), via steps ‘iv’, ‘v’, ‘vi’, and ‘vii’ as:

(9) $$\frac{Q}{A}=J_{\text{norm}}*\left(\frac{\frac{Q}{A}}{E_{\text{TJ}}}\right)*\left(\frac{E_{\text{TJ}}}{J_{v_{i}}}\right)\;=J_{\text{norm}}*1.15\times 10^{-12}*C.$$

Just as current flow through each location of the capillary network wall is directional (into vessel lumen or into interstitial space), so is the resulting volumetric flux. Note that in figure 2(C3), the flow going from the interstitial space into the blood vessels was color coded in red, whereas blue denoted flow going from the vessels into the interstitial space.

Assuming every node (finite element in the capillary wall) in the capillary network model has equal area, the aggregated volumetric flux values across the capillary walls were converted to net fluid exchange per tissue volume by adding up the volumetric flux values at each node and dividing by the volume of the voxel containing the capillary network (figure 2(C3)). This net fluid exchange per tissue volume includes both flows going into and out of the vessels.

We now reach the final stage of the modeling pipeline which predicts the flow per tissue volume across the entire brain for a given tDCS electrode montage and applied current. Recognizing that the pipeline is linear at every scale of model, we repeated the above calculations from step ‘ii’ to ‘viii’ to transform the brain current distribution models in figures 2(B1) and (B2) into flow per tissue volume distribution across the brain as shown in figures 2(C1) and (C2) (figures 2(C3) to (C1), (C2), arrow ‘viii’).

## 3. Results

We developed and applied a novel computational modeling pipeline to predict the generation of interstitial fluid exchange by tDCS assuming all induced flow derives from electroosmosis at TJs. The pipeline is multi-scale spanning a mm-scale tDCS head model (figure 1(A)), and μm-scale capillary network model (figure 1(B)) and a nm-scale BBB TJ model (figures 1(C)–(E)). Electrical current flow predictions started at the head-model to predict the peak current density in a voxel of brain tissue (figures 2(B1) and (B2)). We then considered a capillary network within this brain voxel and predicted current flow around and through the capillary network (figure 2(B3)). For parameter sensitivity, we considered four permutations of a capillary network so that each subsequent step had four possible values. We then modeled current flow within the capillary wall (BBB) itself, including through TJs (figure 2(B6)). At the TJs, the electric field was coupled to fluid flow through electroosmosis (figures 2(C5) and (C6)). The prediction of generating fluid flow was then applied across the entire capillary network (figure 2(C3)). The aggregate fluid flux across the capillary network walls allowed calculation of net fluid exchange in the brain voxel; this yielded a scaling factory from brain electric field to brain net fluid exchange. Using this scaling factor, the fluid exchange across each region of the brain was predicted (figures 2(C1) and (C2)). Thus, this modeling pipeline first moves down scales (from mm, to μm, to nm) in predicting current flow and then back up scales (from nm, to μm, to mm) in predicting fluid flow. This process (involving eight simulations/steps across scale/physics indexed in figure 2 and referred to below) was detailed, along with an underlying experimental parameterization, in Methods.

An MRI-derived tDCS head model (figure 1(A)) with a 1 mA bifrontal pad montage (figure 2(A1)) predicted brain current flow (step ‘i’). Results are consistent with prior simulations (Datta et al 2009, 2012, Brunoni et al 2015) and intra-cranial recordings (Lafon et al 2017, Rahman et al 2017) with diffused current flow between the electrodes, clustered at local peaks. Per mA, the peak electric field in the brain was $∼0.3\;V\;m^{-1}$ corresponding to a peak current density of $0.082\;A\;m^{-2}$ (figures 2(B1), and (B2)). The peak brain current density was applied as a boundary condition to the next scale model of a capillary network (step ‘ii’). The following results should therefore be understood as for the given tDCS head model, and per mA of an applied current.

**Figure 4.**
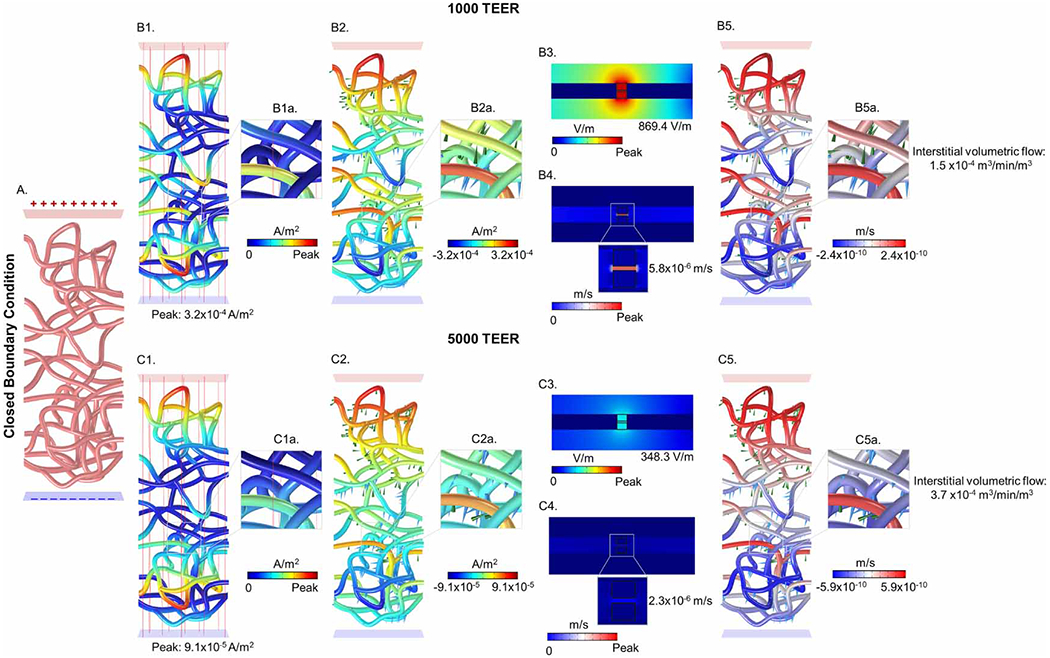
tDCS-induced current flow and coupled fluid exchange across the BBB and tight junction for a *closed* boundary condition at different wall resistivities. (A) illustrates a realistic image derived vasculature network with closed boundary condition. The top panel shows predicted current flow and fluid exchange for the 1000 TEER whereas the bottom panel shows for the 5000 TEER condition. (B1) and (C1) represents current density profiles, (B2) and (C2) current density directionality profiles (red: inward and blue: outward), (B3) and (C3) electric field across the tight junction, (B4) and (C4) fluid exchange across the tight junction, and (B5) and (C5) radial volumetric flux from BBB to brain. Across different wall resistivities, for a closed BC condition, 5000 TEER produced greater interstitial volumetric fluid exchange.

**Figure 5.**
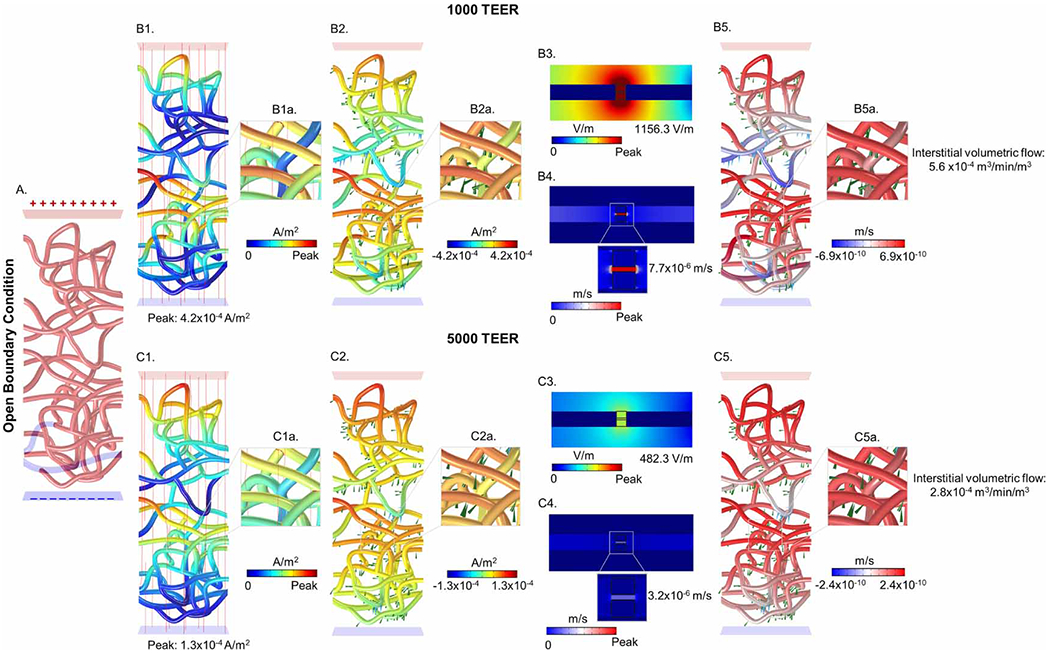
tDCS-induced current flow and coupled fluid exchange across the BBB and tight junction for an *open* boundary condition at different wall resistivities. (A) illustrates a realistic image derived vasculature network with an open boundary condition. The top panel shows predicted current flow and fluid exchange for 1000 TEER whereas the bottom panel shows for 5000 TEER condition. (B1) and (C1) represents current density profile, (B2) and (C2) current density directionality profile (red: inward and blue: outward), (B3) and (C3) electric field across the tight junction, (B4) and (C4) fluid exchange across the tight junction, and (B5) and (C5) radial volumetric flux across the BBB. Across different wall resistivities, for an open BC condition, 1000 TEER produced greater interstitial volumetric fluid exchange.

Inside a brain voxel in the region of peak current density, the current flow through a capillary network was predicted (step ‘iii’). Four conditions were simulated, two boundary conditions and two BBB TEER values. The two boundary conditions were: (1) a ‘closed’ network (figure 4), where all current crossing into the capillary network must exit locally elsewhere in the network; or (2) an ‘open’ network (figure 5) assuming current crossing from brain into local capillaries will be collected through larger vessels to other brain regions. The current density crossing each segment of the capillary network was predicted in either absolute magnitude (figures 4(B1), (B1a), (C1), (Cla) and 5(B1), (B1a), (C1), (C1a)) or considering directionality (figures 4(B2), (B2a), (C2), (C2a) and 5(B2), (B2a), (C2), (C2a)) for both 1000 and 5000 TEERs. The peak current density at the capillary walls (1000 TEER: $3.2\;\times \;10^{-4}\;A\;m^{-2}$ for the closed network and $4.2\;\times \;10^{-4}\;A\;m^{-2}$ for the open network; 5000 TEER: $9.1\;\times \;10^{-5}\;A\;m^{-2}$ for the closed network and $1.3\;\times \;10^{-4}\;A\;m^{-2}$ for the open network) was used as boundary condition to the next scale model of BBB ultrastructure (step ‘iv’).

A vessel cross-section including ultrastructure details (including parenchyma, astrocyte process, astrocytic channel, basement membrane, endothelial cell, interendothelial cleft with TJ channel and TJ strut, surface glycocalyx, and lumen) predicted current flow through the BBB (step ‘v’, figures 2(B6), 4(B3), (C3) and 5(B3), (C3)). The maximum predicted electric field in the TJ for the closed and open vessel networks at 1000 TEER was $869.4\;V\;m^{-1}$ and $1156.3\;V\;m^{-1}$, whereas at 5000 TEER, the maximum electric field in the TJ was $348.3\;V\;m^{-1}$ and $482.3\;V\;m^{-1}$ for the closed and open networks, respectively.

The electric field across the BBB produced electroosmotic-driven fluid flow through the TJ (step ‘vi’). The maximum fluid velocities in the single TJ as a function of the applied E-field were $5.76\;\times \;10^{-6}\;m\;s^{-1}\;\left(E_{TJ}:\;869.4\;V\;m^{-1}\right)$ and $2.30\;\times \;10^{-6}\;m\;s^{-1}\;\left(E_{TJ}:\;348.3\;V\;m^{-1}\right)$ for closed boundary conditions with the 1000 TEER and 5000 TEER, and $7.65\;\times \;10^{-6}\;m\;s^{-1}\;\left(E_{TJ}:\;1156.3\;V\;m^{-1}\right)$ and $3.19\;\times \;10^{-6}\;m\;s^{-1}\;\left(E_{TJ}:\;482.3\;V\;m^{-1}\right)$ for the open boundary condition with the 1000 TEER and 5000 TEER (figures 4 and 5). Note that up to this step, simulations were of electrical current flow with decreases scale (from mm, to μm, to nm). We are now coupling electric current flow (electric fields) to fluid flow, and subsequent steps involve stepping up in scale, starting with the capillary model. The volumetric flux across each capillary wall segment was then calculated analytically (step ‘vii’) by multiplying the product of the proportionality constants from equation (7) and with the current density normal to the wall boundary from the network model (see equation (9)) for different TEERs. Specifically, for a peak current density, the calculated volumetric fluxes were $1.0\;\times \;10^{-9}\frac{m^{3}\;s^{-1}}{\;m^{2}}$ and $1.3\;\times \;10^{-9}\frac{m^{3}\;s^{-1}}{m^{2}}$ at 1000 TEER, and $4.1\;\times \;10^{-10}\;\frac{m^{3}\;s^{-1}}{\;m^{2}}$ and $5.6\;\times \;10^{-10}\;\frac{m^{3}\;s^{-1}}{\;m^{2}}$ at 5000 TEER, for the closed and open vessel networks, respectively. Across the entire capillary network, the volumetric fluxes were $2.4\;\times \;10^{-10}\frac{m^{3}\;s^{-1}}{\;m^{2}}$ and $6.9\;\times \;10^{-10}\frac{m^{3}\;s^{-1}}{\;m^{2}}$ at 1000 TEER, and $5.9\;\times \;10^{-10}\frac{m^{3}\;s^{-1}}{\;m^{2}}$ and $2.4\;\times \;10^{-10}\frac{m^{3}\;s^{-1}}{\;m^{2}}$ at 5000 TEER, for closed and open vessel networks, respectively (figures 4 and 5).

Next, the volumetric fluxes across all segments of the capillary model were translated into net interstitial volumetric fluid exchange $\left({min}^{-1}\right)$. To calculate the net interstitial volumetric fluid exchange (step ‘vii’), the sum of volumetric fluxes per nodes (inward and outward) across the vascular network was first multiplied by the vessel area per node, and then divided by the volume of the voxel containing the vascular network. The net interstitial volumetric fluid exchanges were $1.5\;\times \;10^{-4}\frac{m^{3}\;{min}^{-1}}{\;m^{3}}$ and $5.6\;\times \;10^{-4}\;\frac{m^{3}\;{min}^{-1}}{\;m^{3}}$ at 1000 TEER, and $3.7\;\times \;10^{-4}\frac{m^{3}\;{min}^{-1}}{\;m^{3}}$ and $2.8\;\times \;10^{-4}\frac{m^{3}\;{min}^{-1}}{\;m^{3}}$ at 5000 TEER, for closed and open vessel networks, respectively (figures 4 and 5). Note these values are per mA using the head model/montage indicated and reflected net interstitial volumetric fluid exchange at the brain region of maximal current density.

Thus, the preceding modeling sequence was based on the peak brain current density for an exemplary frontal 1 mA tDCS montage (i.e., the peak brain current density was used as the boundary input for the next model scale and hence implicitly for all subsequent modeling steps). However, each step is linear (current flow, electroosmotic coupling, fluid flow), so that for any given brain current density, all results (including the interstitial fluid enhanced range) scale. Considering the peak brain current density of $0.08\;A\;m^{-2}$ used in the above simulations, the scaling factors from current density to fluid exchange were $1.9\;\times \;10^{-3}\;\left(\;m^{2}(A*min{)}^{-1}\right),\;4.6\;\times \;10^{-3}\;\left(\;m^{2}(A*min{)}^{-1}\right),\;7.0\;\times \;10^{-3}\;\left(m^{2}(A*min{)}^{-1}\right)$, and $\left.3.5\;\times \;10^{-3}\;m^{2}(A*min{)}^{-1}\right)$ for the four conditions (1000 TEER closed, 5000 TEER closed, 1000 TEER open, and 5000 TEER open, respectively).

**Figure 6.**
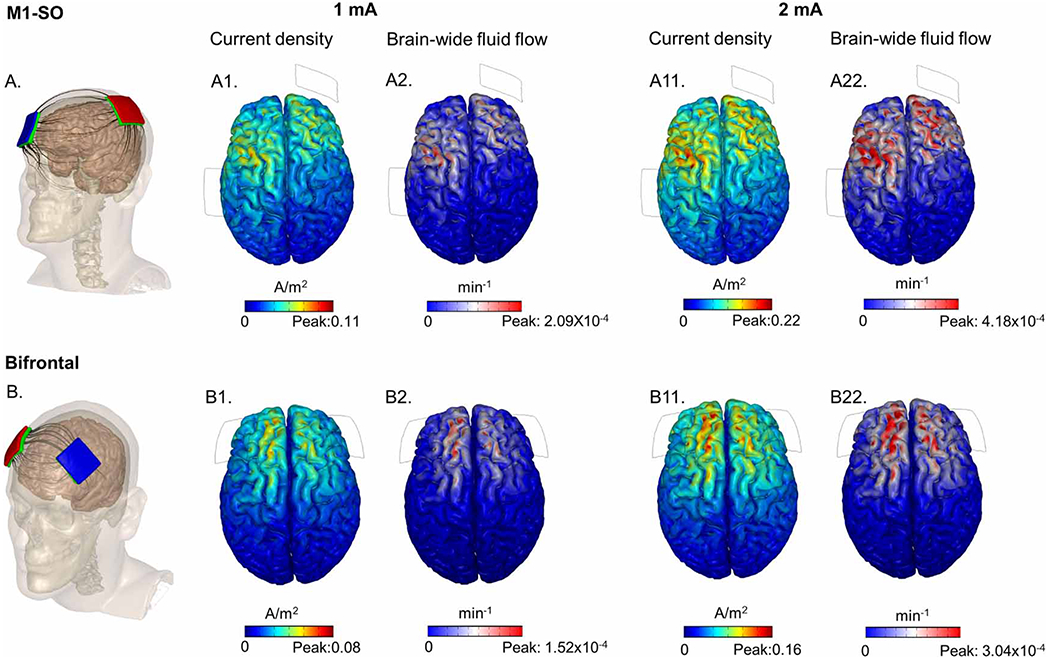
Montage and intensity specific tDCS-coupled brain wide fluid exchange. The top panel shows brain-wide current flow and corresponding fluid exchange at 1 mA and 2 mA intensities for M1-SO montage, whereas the bottom row shows for the bifrontal montage. (A) represents M1-SO tDCS montage, where (A1) shows current density and (A2) shows corresponding brain-wide fluid exchange per 1 mA. (A11) and (A22) represents the current density and corresponding brain-wide fluid exchange per 2 mA. (B) represents bifrontal tDCS montage, where (B1) shows current density magnitude, and (B2) shows corresponding brain-wide fluid flow per 1 mA. (B11) and (B22) represents the current density and corresponding brain-wide flow for 2 mA. Note across montages and intensities, the current density and the field-induced fluid exchange rate scaled linearly. Fluid flow results are assuming closed capillary networks with 1000 TEER.

Finally, figure 6 (step ‘viii’) shows how to apply this scaling factor to predict regional fluid exchange produced by any tDCS dose (electrode position or intensity). We considered two common tDCS electrode placements, M1-S0 and Bifrontal (used in the prior simulations), and two current intensifies, 1 mA (used in the prior simulations) and 2 mA. Each dose produces a brain current density distribution, and a corresponding fluid exchange.

## 4. Discussion

Like all forms of neuromodulation, the conventional theory of tDCS centers on polarization of neurons (Radman et al 2009) leading to changes in brain function and plasticity (Jackson et al 2016, Giordano et al 2017). In this view, the hemodynamic/brain transport changes (Antal et al 2011, Paquette et al 2011, Zheng et al 2011) that follow tDCS are considered ‘epiphenomena’ of neuro-vascular coupling. In contrast, neurovascular-modulation suggests neuromodulation mechanism by direct stimulation of brain transport (Khadka and Bikson 2020, Bahr-Hosseini and Bikson 2021). We previously established that (1) the ultrastructure of the brain vasculature leads to amplification (>400×) of electric fields across the BBB (Khadka and Bikson 2020); and (2) electric fields across an isolated BBB model (i.e., even in absence of neurons) increase water flow by electroosmosis (Cancel et al 2018). The present modeling study aims to relate these effects to the net water exchange boost produced by tDCS; thereby addressing theoretically if tDCS produces meaningful changes in brain clearance mechanisms by directly driving water transport.

The pipeline developed here is novel in several aspects. To our knowledge, this is the first neuromodulation model, for any application, spanning over six orders of magnitude in scale (from cm to nm). As with any staged multi-scale model, we assumed details of finer-scales do not affect results of courser scale simulations; namely microvasculature does not affect brain wide current flow patterns and BBB nanostructure does not impact current patterns across vasculature. While models of electro-osmosis have been presented, to our knowledge this is the first coupling of applied electric fields to brain interstitial water exchange. The quantitative predictions of water exchange by tDCS are novel.

While the process is multi-scale (spanning mm, μm, and nm) and multi-physics (combing electrical and fluid flow physics), given the linearity at each stage, the net result for coupling electrical current flow to fluid exchange in each brain region (voxel) during tDCS is simple. The current density in each voxel (predicted using standard and validated techniques) is coupled to a voxel net fluid exchange by a constant. The details pipeline established here established this constant. We proposed four possible constants based on difference in vascular structure: namely an open or closed network and two values for TEER. Therefore, moving forward any simulation of tDCS current flow can also predict resulting fluid exchange by applying a constant, including models based on open-source software (Huang et al 2019, Saturnino et al 2020) or other solution methods such as boundary element method (BEM) (Brebbia and Partridge 1992, Trozzo et al 2015). Whether a future modeling pipeline can explicitly couple stimulation to BBB polarization and flow at an integrated scale (e.g., using BEM) remains to be shown.

The main predictions of our analysis are that 1 mA applied tDCS current induces interstitial flow rates per unit volume of tissue up to $1.5\;\times \;10^{-4}-5.6\;\times \;10^{-4}\;{min}^{-1}$. These are induced flows in excess (i.e., additive) to the normal background flow. Measurements and estimates of normal background flow are in the range $1\;\times \;10^{-4}-4\;\times \;10^{-4}\;{min}^{-1}$ for human (Hladky and Barrand 2016), rat (Chatterjee et al 2020) and mouse (Wang et al 2017). These estimates suggest roughly a doubling of interstitial flow by 1 mA tDCS. The enhancement would scale increased current levels and vary across head anatomy, brain regions, and tDCS montage (as these factors influence the distribution of electric fields in the brain).

Evidently, our model is sensitive to its underlying assumptions of governing equations and parameters, which were experimentally derived. Individual parameters may factor in one (e.g., open vs closed capillary networks) or multiple (e.g., TEER) scales. A central result of our analysis—that current density per voxel can be linearly related to fluid exchange—is independent of parameters, and sensitivity to parameters can reduce to impact (as we show) on this scaling constant.

Our model predicts increased brain water clearance irrespective of bulk current flow direction. So, any functional differences between current direction (‘cathodal’ vs ‘anodal’, tangential vs radial; (Datta et al 2008, Rahman et al 2013, Evans et al 2022)) would reflect asymmetric vascular anatomy (Secomb et al 2004, Cipolla 2009, Sweeney et al 2018, Bollmann et al 2022) or interaction of vascular with neuronal polarization (Iadecola et al 1997, Drew 2019, Gellner et al 2023). A holistic view of tDCS mechanisms should consider neuronal polarization alongside neurovascular modulation (e.g., district mechanisms engaged depending on dose; (Esmaeilpour et al 2018)). Also, the BBB may respond to a wider range of biphasic waveforms (Pudenz et al 1975, Lopez-Quintero et al 2010, Garcia et al 2012, Arena et al 2014, Rajagopalan et al 2023) with the implications of biphasic current/fluid flux not considered here.

The potential implications of our simulations are compelling. tDCS is broadly used to enhance brain functions; to the extent function is enhanced by boosting (impaired) metabolite deliver/clearance, this provides an explanation for the broad uses of tDCS. tDCS modulates plasticity which underlies lasting therapeutic benefit; plasticity is governed by metabolic capacity (Hamadate et al 2011, Magistretti 2011). Finally, tDCS is trialed for many disorders associated with dysfunctional brain transport and/or pathological accumulation of molecules in the interstitial space (Narita and Yokoi 2017, Agarwal et al 2018, Tsapkini et al 2018, Mishra and Thrasher 2021, da Silva et al 2022, Pilloni et al 2022, Martorella *et al* 2023a). Our models provide a quantitative basis to consider how tDCS may address these pathologies. Ongoing research is warranted to corroborate such a mechanistic link. And such mechanisms are parallel to tDCS direct activation of neurons, or glia, or other pathways.

## Data Availability

All data that support the findings of this study are included within the article (and any supplementary files).
